# The Grade of Dried Jujube (*Ziziphus jujuba* Mill. cv. Junzao) Affects Its Quality Attributes, Antioxidant Activity, and Volatile Aroma Components

**DOI:** 10.3390/foods12050989

**Published:** 2023-02-26

**Authors:** Zhengbao Wu, Shuang Zhang, Lingling Liu, Luyin Wang, Zhaojun Ban

**Affiliations:** 1 Economic Forest Research Institute, Xinjiang Academy of Forestry Sciences, Urumqi 830000, China; 2 Zhejiang Provincial Key Laboratory of Chemical and Biological Processing Technology of Farm Products, School of Biological and Chemical Engineering, Zhejiang University of Science and Technology, Hangzhou 310023, China; 3 Aksu Youneng Agricultural Technology Co., Ltd., Aksu 843001, China

**Keywords:** jujube, grade, mineral elements, antioxidant activity, volatile aroma components

## Abstract

Jujube (*Ziziphus jujuba* Mill. cv. Junzao) has attracted a large number of consumers because it is rich in nutrients, such as carbohydrates, organic acids, and amino acids. Dried jujube is more conducive to storage and transportation, and has a more intense flavor. Consumers are affected by subjective factors, and the most important factor is the appearance of the fruit, including size and color. In this study, fully matured jujubes were dried and divided into five grades according to their transverse diameter and jujube number per kilogram. In addition, the quality attributes, antioxidant activities, mineral elements, and volatile aroma components of dried jujube were further analyzed. As the dried jujube grade increased, the total flavonoid content increased, which was positively correlated with the antioxidant activity. The results showed that small dried jujube had a higher total acidity and lower sugar–acid ratio than large and medium dried jujube, thus, large and medium dried jujube had a better flavor than small dried jujube. However, the antioxidant activity and mineral elements of medium and small dried jujube were superior to large dried jujube. From the edible value analysis of dried jujube, medium and small dried jujube were better than large dried jujube. Potassium is the highest among the measured mineral elements, with contents ranging from 10,223.80 mg/kg to 16,620.82 mg/kg, followed by Ca and Mg. Twenty-nine volatile aroma components of dried jujube were identified by GC–MS analysis. The main volatile aroma components were acids including n-decanoic acid, benzoic acid, and dodecanoic acid. The fruit size affected the quality attributes, antioxidant activity, mineral elements, and volatile aroma components of dried jujube. This study provided a piece of reference information for further high-quality production of dried jujube fruit.

## 1. Introduction

Jujube (*Ziziphus jujuba* Mill.) is a plant of the genus Ziziphus in the family Rhamnaceae. It has been cultivated for a long time and has many varieties in China [1,2,3,4]. Jujube contains numerous essential nutrients, vitamins, and minerals, and also has high medicinal value, with anti-obesity [5], antioxidative [6], antibacterial, and anti-hepatoma activities [7]. Jujube fruit can be eaten not only fresh, but also dried. In addition, jujube is one of the highest yield dried fruits in China [7]. Junzao, as a kind of dried jujube, has been high-profiled due to its high yield, outstanding tolerance, and excellent taste. In 2018, China produced 8.5 million tons of jujube and nearly 5.47 million tons of dried jujube. Among them, Junzao accounted for about 37% of the total dried jujube production [7,8].

The flavor of fruit is perceived mainly through taste and smell [9]. Therefore, the combination of sugars, acids, and volatile aroma compounds in jujube forms the flavor of jujube, which affects the quality of jujube fruit. Jujube fruit contains antioxidant compounds that can ameliorate the oxidative damage caused by free radicals. Previous studies have investigated the antioxidant compounds (such as total phenols, total flavonoids, and ascorbic acid) and antioxidant activities of different tissues of jujube [10].

Drying is an effective method to prevent food from microbial decay, and hot air drying and natural sun drying are traditional drying methods [11]. Among them, hot air drying is the most common drying method in food processing, which is easy to operate and is not affected by climate [11]. Dried jujube is more conducive to storage and transportation, and has a more intense flavor, so it is very popular in the market [12]. Moreover, it possesses abundant nutrition elements and is commonly used as a food ingredient and food seasoning all over the world [8]. Shi et al. found that dried Junzao fruit by hot air was rich in phenolic metabolites and had extensive antibacterial activities [7]. In addition, the drying process altered the composition of phenolic compounds and volatile organic compounds with the fruit aroma [11,13]. Jujube fruit has a unique aroma, which is due to the participation of fruit volatiles in the characterization of fruit aroma characteristics and flavors [14]. The effect of a volatile compound on the final aroma depends on its concentration and the perceived threshold of the specific compound [15]. Previous studies identified aldehydes and acids as the main volatile organic compounds in jujube fruit, such as 2-hexenal, 2-octenal, benzaldehyde, acetic acid, and caproic acid, and low temperature and vacuum drying can retain more volatile aroma components in jujube fruit [16].

Consumers are greatly influenced by subjective factors when purchasing, and fruit appearance phenotype is an important factor, including fruit size, shape, and color [17,18]. However, there are significant differences in the size of jujube fruit under natural conditions, and the correlation between the size and quality of dried jujube has not been reported. At present, the evaluation standard of jujube fruit grade is mainly based on the transverse diameter, longitudinal diameter, single jujube fruit weight, the number of jujube fruit per kilogram, and so on. In this study, we divided the dried jujube into five grades according to their transverse diameter and the jujube number per kilogram, and analyzed the quality attributes, antioxidant activity, mineral elements, and volatile aroma components. The aim of this study was to understand the components of dried jujube and the quality, antioxidant activity, mineral content, and volatile aroma components of different sized dried jujube at the same maturity.

## 2. Materials and Methods

### 2.1. Materials and Treatments

Jujube fruits (*Ziziphus jujuba* Mill. cv. Junzao) were hand-harvested at the commercial maturity stage at a local farm (Xinjiang, China) and dried in a ventilated oven (GZX-9240MBE, Boxun Co., Shanghai, China) at 45 °C for 72 h. According to the transverse diameter and the number of jujube fruits per kilogram, we divided the dried jujube without diseases and pests into five grades (G1, G2, G3, G4, and G5, respectively), as shown in Table 1, and then 500 g of jujube was randomly selected from each group for further experiments. Three biological replications were carried out.

### 2.2. Shape Index and Moisture

The shape index of jujube was the ratio of the longitudinal diameter to the transverse diameter. Moisture content was determined according to the method described by Ajayi et al. [19] with some modifications and was expressed as %. Briefly, 2 g of a jujube fruit sample was dried it in the oven at 105 °C for 2 h, transferred to a desiccator, and cooled for 0.5 h; the above operation was repeated until the sample weight was constant.

### 2.3. Total Soluble Solids (TSS) and Total Acidity (TA)

The jujube fruit were frozen with liquid nitrogen ground to a powder, followed by ultrasonic extraction with distilled water and centrifugation with a centrifuge (5810R, Eppendorf, Hamburg, Germany). TSS was determined by reference to Gao et al. [20] with some modifications. TA was determined based on previously reported methods with some modifications and was expressed as g/kg [21,22].

### 2.4. Total Phenolics (TP) and Total Flavonoids (TF)

After grinding in liquid nitrogen, we weighed 1 g of the sample and added 25 mL 75% ethanol solution to the extract for 60 min. The TP content was determined using the Folin–Ciocalteu procedure described by Jiménez-Muñoz et al. [23] with some modifications. Absorbance was measured with a microplate reader (Infinite M200 Pro, Tecan, Männedorf, Switzerland) at 765 nm. The TP concentration in the sample was calculated by drawing a standard curve with the gallic acid standard. The result of the TP was expressed based on the fresh weight as g/kg. The TF content was determined according to the method described by Kou et al. [24] with some modifications. Absorbance was measured with a microplate reader at 500 nm. The TF concentration in the samples was calculated by drawing a standard curve with the rutinum standard and was expressed as g/kg.

### 2.5. Ascorbic Acid (AsA)

The analysis of the AsA content was determined according to the 2,6-dichlorophenolindophenol method described by Kou et al. [24] with some modifications, and was presented in mg/kg. We added 10 mL of 2% oxalic acid solution to 1 g of the jujube fruit sample and ground it to a homogenate. We diluted 5 mL of filtered solution with 2% oxalic acid to 50 mL, took 10 mL of the solution, and titrated it with 0.2 g/L 2,6-dichlorophenolindophenol to the endpoint. The 2,6-dichlorophenolindophenol solution was calibrated with 1 mg/mL AsA standard solution.

### 2.6. Cyclic Adenosine Monophosphate (cAMP)

The content of cAMP was determined with the high performance liquid chromatography method [21]. The 5 g jujube fruit samples were extracted by adding 20 mL of methanol/0.05 mol/L monopotassium phosphate solution (1/4, *v*/*v*) for 40 min, and centrifuged. Then, the supernatant was passed through a 0.22 μm water-phase filter membrane, and assessed by high performance liquid chromatograph (Waters e2695, Waters, Milford, MA, USA).

### 2.7. Antioxidant Activity

A sample extract was obtained by weighing 0.3 g of sample powder, adding 10 mL of 50% methanol solution, and extracting for 30 min [25,26]. The analyses of the free hydrophilic antioxidant fraction were conducted using the 2,2-diphenyl picrylhydrazyl (DPPH) method, whereas the bound antioxidant fraction was analyzed with the ferric reducing antioxidant power (FRAP) method. The result of DPPH radical scavenging activity was expressed as a %. The result of FRAP was expressed as g of Trolox equivalents (TE)/kg.

### 2.8. Mineral Elements

The contents of potassium (K), calcium (Ca), magnesium (Mg), zinc (Zn), and copper (Cu) in jujube fruit were determined by atomic absorption spectrometry (pinAAcle900T, Perkin Elmer, Massachusetts, USA) according to the method described by Mattila et al. [27] with some modifications, and were presented in mg/kg. After digestion, the absorbance of the samples was determined at 766.5 nm, 422.7 nm, 285.2 nm, 213.9 nm, and 324.8 nm by atomization. The absorbance value of mineral elements within the right concentration range was positively proportional to the content of mineral elements, and the content was determined by comparing with the standard series ratio.

### 2.9. Volatile Aroma Components Analysis

The method of analysis and determination of volatile aroma components of dried jujube was modified with reference to Bi et al. [21]. The determination was carried out by solid phase microextraction (SPME, AOC5000, CTC Analytics AG, Zwingen, Switzerland) and gas chromatography–mass spectrophotometry (GC–MS, QP2010Plus, Shimadzu, Tokyo, Japan). The volatile aroma components of the sample were exacted with 50/30 μm divinylbenzene/carboxen/polydimethylsiloxane (DVB/CAR/PDMS) fibers, then used for GC–MS analysis. The injection temperature was 250 °C, the carrier gas was Helium, and the flow rate was 1.0 mL/min. The column temperature was held at 50 °C for 5 min, and the temperature was increased to 150 °C at a rate of 3 °C/min, then increased to 250 °C at a rate of 10 °C/min and maintained at 250 °C for 2 min.

### 2.10. Statistical Analysis

All experiments were performed with three biological replications and three technical replications. Results are presented as mean ± standard deviation (SD). One way analysis of variance (ANOVA) and the Duncan’s test were used to determine the differences among the means. Correlations between parameters were examined using the Pearson correlation. Differences were considered statistically significant at *p* < 0.05.

## 3. Results and Discussion

### 3.1. The Quality Attributes of Dried Jujube

**Figure 1:**
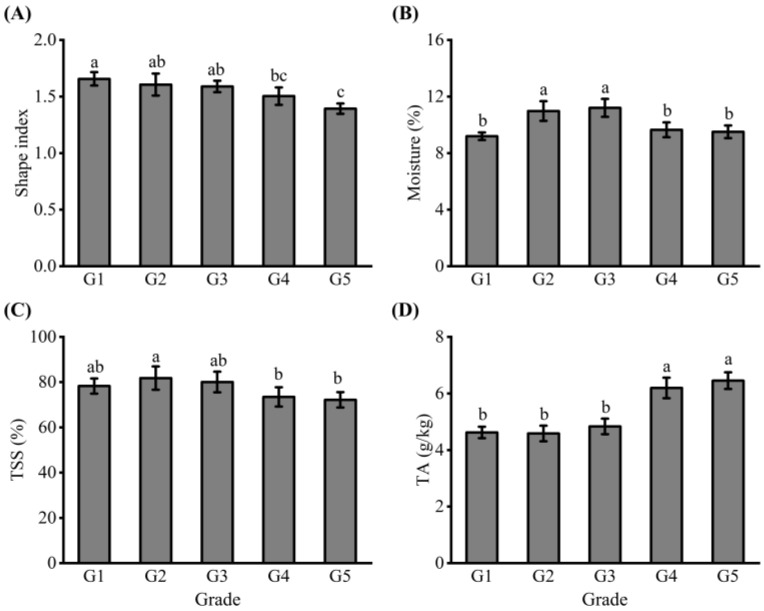
The quality attributes of dried jujube. The analysis of (**A**) shape index, (**B**) moisture, (**C**) total soluble solids (TSS), and (**D**) total acidity (TA) of dried jujube. The dried jujubes were arranged in the order from large to small, which were G1, G2, G3, G4, and G5 groups, respectively. Data are expressed as means ± standard deviation (SD) from three replications. Means with different letters are significantly different (*p* < 0.05).

Fruit shape index is one of the important commodity quality indexes [28]. As the grade of dried jujube increased, the fruit shape index decreased from 1.66 to 1.39 and gradually approached 1, which indicated that the shape of small dried jujube was more circular than that of medium and large dried jujube (Figure 1A). After the same drying conditions, the moisture content of the five grades of jujube was different. In Figure 1B, the moisture content of dried jujube in G1 group was the lowest (9.2%), but it had no significant difference with that in the G4 and G5 (*p* > 0.05) groups. The moisture content of dried jujube seriously affected the quality and shelf life. Our results indicated that compared with medium dried jujube, large and small dried jujube were more favorable for long-term storage, especially the G1 group.

TSS and TA content determine the taste and flavor of dried jujube. In Figure 1C, The TSS content of dried jujube ranged from 81.8% to 72.2%, and the smallest jujube (G5) had the lowest TSS content. However, The TA content of dried jujube increased from 4.58 g/kg to 6.45 g/kg with the increase of grade from G1 to G5 (Figure 1D). The sugar–acid ratios the of five grades if dried jujube were 169.4%, 178.4%, 165.6%, 118.6%, and 111.9%, respectively. These indicated that dried jujube of G2 have a sweeter texture and a more intense flavor.

Li et al. [29] studied the five cultivars of Chinese jujube and pointed out that the sugar content and composition of jujube with different varieties and growing environments vary widely. Chen et al. [22] also proposed that the TSS of fresh Junzao fruit from four different cultivation districts varied from 27.2% to 30.6%. However, the TSS of dried jujube was even higher. The trend of moisture content in dried jujube was similar to that of the TSS content, which indicates that moisture content in dried jujube may be related to TSS content. When the content of TSS is low, and the water in the fruit is easy to spread during drying, and the drying efficiency is high, the moisture content is low. In addition, except the G1 group, the change trend of moisture content in the other four groups was the same as that of fruit shape index, which indicated that the drying effect was related to fruit shape, and the grade of dried jujube affected the moisture content. Khalid et al. [30] found that mandarin fruit size is inversely proportional to TA content, which is similar to our conclusion. Similarly, they pointed out that a lower sugar–acid ratio is recorded in small sized fruit in contrast to medium and large sized fruit [30].

### 3.2. TP and TF of Dried Jujube

**Figure 2:**
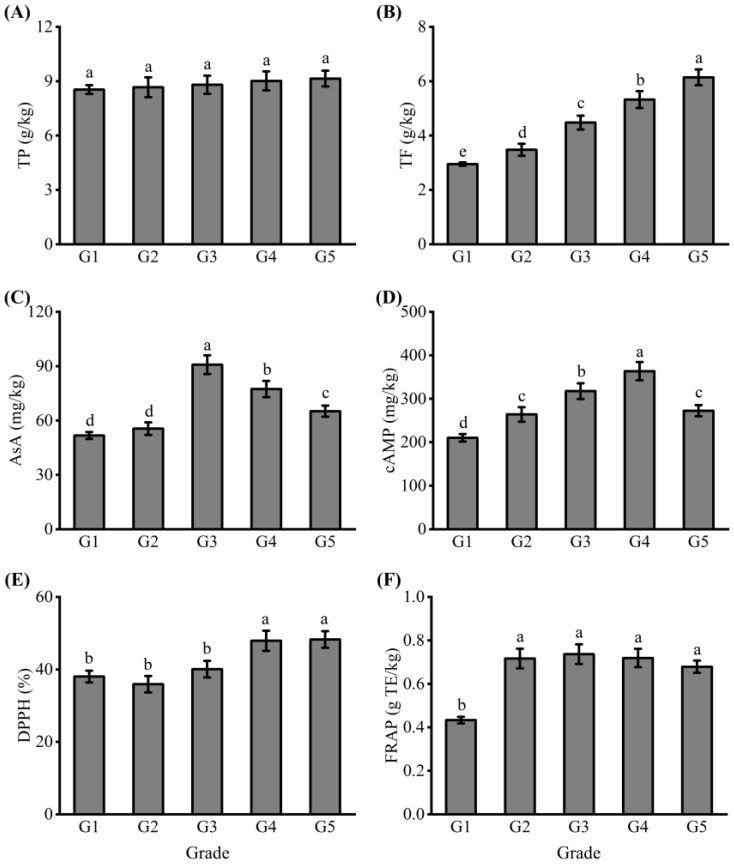
The contents of (**A**) total phenolics (TP), (**B**) total flavonoids (TF), (**C**) ascorbic acid (AsA), (**D**) cyclic adenosine monophosphate (cAMP), (**E**) 2,2-diphenyl picrylhydrazyl (DPPH) radical scavenging activity, and (**F**) ferric reducing antioxidant power (FRAP) of dried jujube. The dried jujube were arranged in the order from large to small, which were G1, G2, G3, G4, and G5 groups, respectively. Data are expressed as means ± standard deviation (SD) from three replications. Means with different letters are significantly different (*p* < 0.05).

Phenolics are the most common secondary metabolites in fruit, and they influence the quality, color, and flavor of fruit [31]. The composition and content of phenolics in fruit varies with the variety, texture, and processing of fruit. In Figure 2A, the TP content of dried jujube in different grades was similar, ranging from 8.54 g/kg to 9.14 g/kg. Wojdyło et al. [32] found 25 phenolic compounds in Spanish jujube, and total phenolic compounds (especially polymer proanthocyanin and quercetin derivatives) and ascorbic acid contributed significantly to the antioxidant capacity of jujube. The growth and development of jujube affected the TP content [20,33]. The maturity of different graded jujube was the same, which may be the reason for the little difference in the TP content. Barbagallo et al. [34] analyzed the TP content of grapes with different fruit size and found that the TP content decreased with grape weight and was affected by environment, region, climate, and other factors, thus, the phenomenon of TP content variation in this experiment could be explained.

Flavonoids are phenolic compounds that are widely found in fruits and are particularly important for human health [10,35]. Jujube is rich in flavonol glycosides composition, and the difference in variety and maturity can lead to differences in the flavonoid content in jujube [20,36,37,38]. In Figure 2B, the TF content of dried jujube increased from 2.95 g/kg to 6.14 g/kg with the increase in grade of G1 to G5. These results indicated that fruit enlargement accelerated the consumption and decreased the accumulation of flavonoids. Barbagallo et al. [34] pointed out that the TF amount increased with grape size, which was similar to our results. TP and TF contents of fruit in different parts was also different. Zhang et al. [10] found that the peel of all jujube species had the highest antioxidant capacity, reflecting the highest levels of total phenols, flavonoids, and anthocyanins in peel.

### 3.3. AsA and cAMP of Dried Jujube

AsA has the effect of antioxidation and scavenging of free radicals, which widely exists in fruit and vegetables, and has a high content in jujube [24]. In Figure 2C, the AsA content of dried jujube increased gradually from G1 to G3, and then decreased. AsA content ranged from 51.71 mg/kg to 90.80 mg/kg, which was higher than that of pear-jujube at different ripening stages, as previously reported. The AsA content of G3 was the highest, being 1.76 times that of G1. This conclusion was partly similar to the findings of Wu et al. [39], who found that the AsA content decreased during pear-jujube ripening. In addition, the results of AsA and moisture were similar, which may be related to the fact that AsA is a water-soluble and heat-sensitive compound [40].

cAMP is a physiologically active substance involved in the regulation of material metabolism and biological functions in cells, and plays an important role in the regulation of sugar, fat metabolism, nucleic acids, and protein synthesis. In Figure 2D, the cAMP content of dried jujube increased gradually from G1 to G4, but the cAMP content of G5 decreased. The cAMP content in different grades of dried jujube reached the peak at G4 (363.55 mg/kg), and the cAMP content of G1 of dried jujube was the lowest (210.26 mg/kg). The cAMP content of dried jujube was similar to that of moisture content and AsA content, except at the highest point. The cAMP content of jujube fruit was higher than that of most fruits [24]. There were significant differences in the cAMP content of different cultivars and cultivation areas in jujube fruit. Zhang et al. [6] pointed out that many factors, such as sample collection period and extraction method, would affect the content of cAMP, which might lead to the results of our experiment. Chen et al. [22] found that the cAMP content of fresh Junzao jujube in the Kashi district was the lowest (50.31 mg/kg), while that in the Aksu district was 87.90 mg/kg. Different processing methods can also affect the cAMP content of jujube. Previous studies have shown that the cAMP content of dried jujube increases while that of steamed jujube decreases [41].

### 3.4. The Antioxidant Activity of Dried Jujube

Free radicals induce the oxidation of lipids, proteins, and DNA, which can lead to adverse events, thus, free radical scavenging is one of the important functions of antioxidants [24]. We quantified the antioxidant activity of five grades of dried jujube by DPPH and FRAP methods. In Figure 2E, the DPPH radical scavenging activity of dried jujube increased with grades, and the difference between groups G3 and G4 was more pronounced, with a 7.84% increase in DPPH radical scavenging activity in G4. The DPPH radical scavenging activities of G4 and G5 were higher than those of the other three groups. In Figure 2F, the FRAP of dried jujube also increased with grades, and the FRAP of G1 was the lowest in the five grades at 0.43 g TE/kg. These results indicated that the small dried jujube had a higher antioxidant activity measured from the DPPH method, and medium and small dried jujube had higher antioxidant activity measured from the FRAP method. The change trend was similar to the TF content, and the accumulation of flavonoids increased the antioxidant activity of dried jujube. This conclusion was similar to Li et al. [26], who point out that DPPH radical scavenging activity and FRAP were positively correlated with rutin and other flavonoid metabolites through correlation analysis.

### 3.5. The Mineral Elements of Dried Jujube

The results of the analysis of the mineral contents of dried jujube were summarized in Table 2. Potassium regulates intracellular osmotic pressure and the acid–base balance of body fluids, and is also involved in the metabolism of intracellular sugars and proteins. In addition, potassium was the predominant mineral in the five grades of dried jujube. The K contents ranged from 10,223.80 mg/kg to 16,620.82 mg/kg, and the richest source of K in this study was G5. Calcium helps to lower blood pressure, regulate the nervous system, and participate in muscle contraction. From the statistical analysis, the Ca content of the five grades of dried jujube were significantly different (*p* < 0.05), and the richest source of Ca in this study also was G5. Magnesium is an activator of enzymes, which participates in the normal life activities and metabolic processes of organisms. The Mg contents ranged from 206.35 mg/kg to 253.37 mg/kg, and the richest source of Mg in this study was G4. The five grades of dried jujube contained relatively low amounts of Zn and Cu, which are important because Zn is nutritionally essential for all organisms and Cu participates in numerous enzyme-catalyzed oxidation–reduction reactions and processes [29]. Regarding Zn content, the highest value was found in G3 (4.10 mg/kg), followed by G5 (3.75 mg/kg), and G4 (3.63 mg/kg). There were also significant differences in the Cu content of the five grades of dried jujube, in which the highest Cu content was found in G5 (3.06 mg/kg) and the lowest Cu content was found in G3 (1.70 mg/kg).

The content of mineral elements is influenced by many factors, such as jujube cultivar, development stage, soil, and climate in the cultivation area. Li et al. [29] measured the mineral content of five cultivars of jujube in their experiment, and found that the Ca content of Junzao jujube (1179.88 mg/kg) was higher than that of the other four cultivars of jujube (Jinsixiaozao, Yazao, Jianzao and Sanbianhong), but Mg content was the lowest (246.12 mg/kg).

### 3.6. The Correlation Analysis of Dried Jujube

In order to understand more fully the correlation between the quality attributes, antioxidant activity, and mineral elements, we carried out a correlation analysis of the above indicators. In Table 3, a significant positive correlation between shape index and TSS content was noted in our results (R^2^ = 0.526, *p* < 0.05). However, the shape index was negatively correlated with some indicators (TA, R^2^ = −0.777, *p* < 0.01; TF, R^2^ = −0.808, *p* < 0.01; DPPH, R^2^ = −0.720, *p* < 0.01; K, R^2^ = −0.640, *p* < 0.05; Ca, R^2^ = −0.748, *p* < 0.01; Zn, R^2^ = −0.569, *p* < 0.05; Cu, R^2^ = −0.710, *p* < 0.01). The results showed that TSS content increased with the increase in the ratio of the longitudinal diameter to transverse diameter, but the antioxidant activities and mineral contents decreased. Therefore, the taste of large dried jujube is better than that of small dried jujube, but the quality of small dried jujube is better than that of large dried jujube, which is similar to previous studies [30,34]. Barbagallo et al. [34] pointed out that the fruit composition varies greatly with the fruit size, and the influence of fruit size should be considered, as well as the influence of other environmental factors, on fruit composition.

Our results indicated a positive correlation between antioxidant activity and TF (DPPH, R^2^ = 0.912, *p* < 0.01; FRAP, R^2^ = 0.575, *p* < 0.05), which was consistent with other reported results [6,26]. DPPH radical scavenging activity also was highly positively correlated with TA, cAMP, and mineral element contents (TA, R^2^ = 0.982, *p* < 0.01; cAMP, R^2^ = 0.597, *p* < 0.05; K, R^2^ = 0.921, *p* < 0.01; Ca, R^2^ = 0.647, *p* < 0.01; Mg, R^2^ = 0.593, *p* < 0.05; Zn, R^2^ = 0.619, *p* < 0.05; Cu, R^2^ = 0.697, *p* < 0.01). In addition, the FRAP was positively correlated with moisture, AsA, cAMP, and some mineral element contents (moisture, R^2^ = 0.674, *p* < 0.01; AsA, R^2^ = 0.658, *p* < 0.01; cAMP, R^2^ = 0.786, *p* < 0.01; Mg, R^2^ = 0.654, *p* < 0.01; Zn, R^2^ = 0.845, *p* < 0.01).

### 3.7. The Volatile Aroma Components of Dried Jujube

The main volatile aroma components of dried jujube are shown in Table 4; among these, the highest volatile aroma components of dried jujube were acids (n-decanoic acid, benzoic acid, and dodecanoic acid). In Table 4, the relative content of benzoic acid, n-decanoic acid, dodecanoic acid, benzoic acid, and octanoic acid were the highest among the five grades of dried jujube (up to 17.19%, 16.37%, 18.85%, 22.63%, and 17.94%, respectively). The relative content of 2-octenoic acid, methyl (Z)-9-hexadecenoic acid, methyl dodecanoic acid, and methyl myristoleate of the G2 dried jujube reached the maximum in the five grades. Among the five grades of dried jujube, the relative contents of five volatile aroma components (1-octadecene, dibutyl phthalate, dodecanoic acid, hydrocinnamic acid, octadecane) reached a peak in the G3 dried jujube, and the relative contents of the other five volatile aroma components (acetic acid, benzaldehyde, heptanoic acid, nonanoic acid, and octanoic acid) were the highest in the G5 dried jujube. In addition, the relative contents of 6,10,14-trimethyl-2-pentadecanone and benzoic acid of the G4 dried jujube were the highest in the five grades. This was similar to the previous conclusion [42,43]. However, Wang et al. [44] identified 31, 31, 32, and 32 volatile aroma components from Tangzao, Muzao, Lizao, and Qingrunhongzao, respectively, and pointed out that aldehydes were the main volatile aroma components in jujube. Bi et al. [21] found that after drying, the relative content of aldehydes in jujube decreased, but the content of alkanes and ketones increased. The volatile aroma components of dried jujube are very complex. Spadafora et al. [45] pointed out that many factors, such as fruit maturity, storage temperature, and processing process, can affect the volatile aroma components. These may account for the results.

## 4. Conclusions

Consumers are influenced by subjective factors when buying dried jujube, and the most important factor is the fruit appearance. This study found that fruit size had effects on quality attributes, antioxidant activity, mineral elements, and volatile aroma components of dried jujube. From the flavor analysis of dried jujube, the flavor of large and medium dried jujube was better than that of small dried jujube. The correlation analysis showed that the TF content of dried jujube was positively correlated with antioxidant activity, and AsA content was positively correlated with cAMP content. However, from the analysis of edible value, medium and small dried jujube had a higher antioxidant capacity. Potassium, as an essential element in organisms, was the highest of the measured mineral elements of dried jujube. The volatile aroma components of dried jujube were complex, among which the relative content of acid was the highest. This study provided a reference for the evaluation of dried jujube fruit quality and the improvement of grading standards, which aimed to improve the commercial value and market competitiveness of dried jujube.

## Figures and Tables

**Table 1:** The grading standard of dried jujube.

Grade	G1	G2	G3	G4	G5
Transverse diameter (mm)	≥32	≥30, <32	≥28, <30	≥26, <28	≥24, <26
Number of jujube per kilogram	≤70	71–85	86–105	106–125	126–150

**Table 2:** The mineral element contents of dried jujube.

Grades	Contents (mg/kg)				
	K	Ca	Mg	Zn	Cu
G1	13,485.13 ± 393.24 b	149.89 ± 5.45 d	206.35 ± 9.22 b	0.88 ± 0.03 d	1.97 ± 0.06 b
G2	10,223.80 ± 648.03 a	174.40 ± 11.05 c	222.01 ± 14.06 b	2.30 ± 0.14 c	1.80 ± 0.12 bc
G3	12,308.21 ± 701.14 b	196.10 ± 11.15 b	225.97 ± 13.00 b	4.10 ± 0.23 a	1.70 ± 0.09 d
G4	15,442.79 ± 895.98 a	177.62 ± 10.33 bc	253.37 ± 14.63 a	3.63 ± 0.21 b	2.01 ± 0.12 b
G5	16,620.82 ± 788.23 a	308.67 ± 14.64 a	216.99 ± 10.31 b	3.75 ± 0.17 b	3.06 ± 0.15 a

The dried jujube were arranged in the order from large to small, which were G1, G2, G3, G4, and G5 groups, respectively. Data are expressed as means ± standard deviation (SD) from three replications. Means with different letters are significantly different (*p* < 0.05).

**Table 3:** The correlation analysis of dried jujube.

	Shape Index	Moisture	TSS	TA	AsA	cAMP	TP	TF	DPPH	FRAP	K	Ca	Mg	Zn	Cu
Shape index	1														
Moisture	0.236	1													
TSS	0.526 *	0.776 **	1												
TA	−0.777 **	−0.284	−0.491	1											
AsA	−0.202	0.48	0.056	0.288	1										
cAMP	−0.335	0.344	−0.068	0.537 *	0.811 **	1									
TP	−0.401	0.318	0.269	0.697 **	0.396	0.512	1								
TF	−0.808 **	−0.081	−0.485	0.928 **	0.499	0.611 *	0.638 *	1							
DPPH	−0.720 **	−0.249	−0.434	0.982 **	0.405	0.597 *	0.734 **	0.912 **	1						
FRAP	−0.374	0.674 **	0.144	0.377	0.658 **	0.786 **	0.502	0.575 *	0.36	1					
K	−0.640 *	−0.525 *	−0.542 *	0.899 **	0.184	0.29	0.581 *	0.773 **	0.921 **	−0.011	1				
Ca	−0.748 **	−0.065	−0.349	0.709 **	0.159	0.106	0.528 *	0.817 **	0.647 **	0.352	0.621 *	1			
Mg	−0.219	0.337	0.16	0.535 *	0.585 *	0.898 **	0.679 **	0.468	0.593 *	0.654 **	0.31	−0.013	1		
Zn	−0.569 *	0.373	−0.204	0.595 *	0.838 **	0.804 **	0.501	0.822 **	0.619 *	0.845 **	0.357	0.573 *	0.546 *	1	
Cu	−0.710 **	−0.4	−0.448	0.769 **	−0.152	−0.077	0.504	0.716 **	0.697 **	0.028	0.780 **	0.898 **	−0.059	0.258	1

TSS, Total soluble solids; TA, total acidity; TP, total phenolics; TF, total flavonoids; AsA, ascorbic acid; cAMP, cyclic adenosine monophosphate; DPPH, 2,2-diphenyl picrylhydrazyl radical scavenging activity; FRAP, ferric reducing antioxidant power. * *p* < 0.05; ** *p* < 0.01; ns, not significant.

**Table 4:** The analysis of volatile aroma compounds of dried jujube.

Compounds	Relative Content (%)					Evolution
	G1	G2	G3	G4	G5	
1-Octadecene	0.79 ± 0.04 cd	1.05 ± 0.13 bc	2.18 ± 0.15 a	1.29 ± 0.33 b	0.61 ± 0.1 d	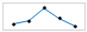
1-Pentadecene	0.35 ± 0.08 a	0.4 ± 0.01 a	0.2 ± 0.04 b	0.37 ± 0.06 a	0.33 ± 0.03 a	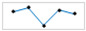
2-Octenoic acid	2.66 ± 0.04 b	3.31 ± 0.11 a	2.36 ± 0.32 d	2.51 ± 0.01 bc	2.66 ± 0.03 b	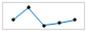
2-Pentadecanone, 6,10,14-trimethyl-	0.59 ± 0.04 c	0.96 ± 0.05 b	0.95 ± 0.08 b	1.26 ± 0.2 a	0.52 ± 0.22 c	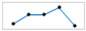
2-Undecanone, 6,10-dimethyl-	0.85 ± 0.19 b	1.16 ± 0.08 a	0.37 ± 0.13 c	0.91 ± 0.13 ab	0.99 ± 0.17 ab	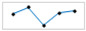
9-Hexadecenoic acid, methyl ester, (Z)-	0.40 ± 0.05 c	1.30 ± 0.13 a	0.76 ± 0.08 b	0.19 ± 0.04 d	0.20 ± 0.08 d	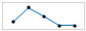
Acetic acid	8.13 ± 1.06 b	6.38 ± 0.8 c	5.23 ± 0.68 cd	4.66 ± 0.87 d	10.88 ± 0.87 a	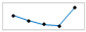
Benzaldehyde	0.52 ± 0.05 b	0.39 ± 0.05 c	0.28 ± 0.07 c	0.38 ± 0.03 c	1.37 ± 0.12 a	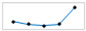
Benzoic acid	17.19 ± 2.47 b	13.23 ± 0.29 c	17.07 ± 1.13 b	22.63 ± 1.49 a	0 ± 0 d	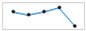
Benzoic acid, 2-ethylhexyl ester	0.55 ± 0.12 a	0 ± 0 b	0.66 ± 0.24 a	0.43 ± 0.14 a	0.15 ± 0.06 b	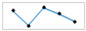
Decanoic acid, ethyl ester	0.71 ± 0.11 b	0.92 ± 0.03 b	1.39 ± 0.3 a	1.42 ± 0.24 a	0 ± 0 c	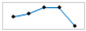
Dibutyl phthalate	0.30 ± 0.03 b	0.34 ± 0.04 b	1.12 ± 0.34 a	0.22 ± 0.06 b	0.12 ± 0.05 b	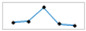
Dodecanoic acid	11.99 ± 1.23 bc	12.25 ± 1.3 b	18.85 ± 2.37 a	8.72 ± 1.69 cd	7.83 ± 2.15 d	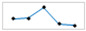
Dodecanoic acid, methyl ester	1.82 ± 0.52 b	2.38 ± 0.11 a	0.67 ± 0.36 c	1.39 ± 0.12 b	1.33 ± 0.09 b	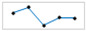
Ethyl 9-hexadecenoate	1.33 ± 0.13 b	2.02 ± 0.18 a	1.73 ± 0.24 a	0.27 ± 0.07 c	0.40 ± 0.15 c	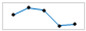
Heptadecane	0.57 ± 0.17 a	0 ± 0c	0.70 ± 0.13 a	0.51 ± 0.12 ab	0.31 ± 0.04 b	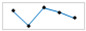
Heptanoic acid	5.78 ± 0.52 bc	4.8 ± 0.21 c	2.55 ± 0.57 d	7.66 ± 0.52 b	10.17 ± 2.21 a	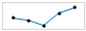
Hexadecane	0.60 ± 0.15 b	0.61 ± 0.02 b	0 ± 0 c	1.11 ± 0.14 a	1.36 ± 0.24 a	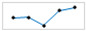
Hexadecane, 2,6,10,14-tetramethyl-	0.54 ± 0.14 ab	0 ± 0 c	0.73 ± 0.20 a	0.50 ± 0.21 ab	0.26 ± 0.07 bc	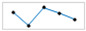
Hexadecanoic acid, methyl ester	0.26 ± 0.02 ab	0.30 ± 0.02 a	0.23 ± 0.04 bc	0.18 ± 0.03 c	0.06 ± 0.02 d	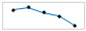
Hexanoic acid	6.75 ± 0.44 ab	5.73 ± 0.44 b	2.88 ± 0.38 c	5.75 ± 0.55 b	7.56 ± 1.66 a	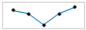
Hydrocinnamic acid	0.76 ± 0.08 b	0.76 ± 0.02 b	2.03 ± 0.4 a	0.83 ± 0.05 b	0.50 ± 0.04 b	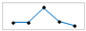
Methyl myristoleate	0.90 ± 0.08 b	1.47 ± 0.1 a	0.47 ± 0.08 c	0.33 ± 0.06 cd	0.32 ± 0.09 d	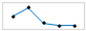
n-Decanoic acid	14.35 ± 0.69 ab	16.37 ± 0.71 ab	16.57 ± 1.69 a	13.28 ± 0.83 b	15.10 ± 2.83 ab	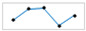
Nonanoic acid	1.65 ± 0.05 b	1.47 ± 0.03 c	0.91 ± 0.07 d	1.72 ± 0.06 b	1.87 ± 0.04 a	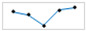
Octadecane	0.47 ± 0.09 b	0 ± 0 d	0.68 ± 0.06 a	0.41 ± 0.1 b	0.21 ± 0.06 c	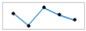
Octanoic acid	4.55 ± 1.04 b	5.24 ± 0.52 b	1.91 ± 0.39 c	5.52 ± 0.44 b	17.94 ± 0.28 a	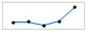
Pentadecane	0.61 ± 0.09 a	0.48 ± 0.02 ab	0.44 ± 0.13 b	0.46 ± 0.08 ab	0.55 ± 0.02 ab	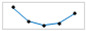
Pentadecane, 2,6,10,14-tetramethyl-	0.63 ± 0.21 a	0 ± 0 b	0.68 ± 0.28 a	0.49 ± 0.19 a	0.33 ± 0.15 ab	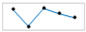

The dried jujube were arranged in the order from large to small, which were G1, G2, G3, G4, and G5 groups, respectively. Data are expressed as means ± standard deviation (SD) from three replications. Means with different letters are significantly different (*p* < 0.05).

## Data Availability

Data is contained within the article.
